# Epidemiological Survey of Porcine Circovirus Type 2 (PCV2) in Large-Scale Pig Farms in Hubei Province and Comprehensive Evaluation of Commercial Vaccine Efficacy

**DOI:** 10.3390/vaccines13101066

**Published:** 2025-10-18

**Authors:** Wenjun Liao, Zhaofang Xi, Rui Fang, Bang Shen, Junlong Zhao

**Affiliations:** 1 State Key Laboratory of Agricultural Microbiology, Huazhong Agricultural University, Wuhan 430070, China; liaowenjun_123@webmail.hzau.edu.cn (W.L.); fangrui19810705@163.com (R.F.); shenbang@mail.hzau.edu.cn (B.S.); 2 Chia Tai Group (Central South) Research Institute, Xiangyang 441111, China; zfxi1982@163.com

**Keywords:** porcine circovirus type 2, epidemiology, vaccine efficacy, subunit vaccine, economic benefit

## Abstract

Background: Porcine circovirus type 2 (PCV2) is the primary pathogen responsible for postweaning multisystemic wasting syndrome (PMWS) and related diseases, leading to significant economic losses in the global pig industry. Methods: This study conducted a thorough epidemiological survey between 2022 and 2024, gathering 6600 samples from 24 large-scale pig farms in Hubei Province. On the basis of these findings, the immune response and economic benefits of two representative commercial PCV2 subunit vaccines, recombinant baculovirus CP08 and Ingelvac CircoFLEX^®^, were assessed in a modern fattening farm in Xiangyang city. Results: The results indicated no detection of viral antigens in sows; however, weaned piglets and fattening pigs presented high positivity rates, with 8-week-old nursery pigs identified as the peak period for infection. Both vaccines significantly improved average weight gain and reduced antigen positivity, with Ingelvac CircoFLEX^®^ demonstrating superior viral control and economic returns. Conclusions: This study offers valuable scientific and practical guidance for PCV2 control strategies and vaccine selection in Hubei and comparable regions.

## 1. Introduction

Porcine circovirus type 2 (PCV2) is among the most significant viruses impacting the global swine industry, posing serious threats to swine health and resulting in considerable economic losses [1]. These symptoms include respiratory distress, diarrhea, encephalitis, congenital tremors, reduced daily weight gain, anemia, jaundice, dermatitis, and nephritis [2,3]. Collectively, these conditions are classified as porcine circovirus-associated disease (PCV-AD) [4,5,6].

PCV2 is a small, non-enveloped, single-stranded circular DNA virus that belongs to the genus Circovirus within the Circoviridae family. It ranks among the smallest known animal viruses, measuring only 16–18 nm in diameter and possessing a genome approximately 1700 bases in length [7,8]. First identified in the late 1990s [8,9], the genome of PCV2 contains approximately 10 open reading frames (ORFs), with ORF1 and ORF2 coding for the replicase and capsid proteins, which are essential for viral replication [10,11]. Notably, although PCV2 is a DNA virus, it has the highest nucleic acid substitution rate recorded among DNA viruses, at approximately 1.2 × 10^−3^ substitutions per site per year [12,13].

Analyses of the whole genome and the ORF2 gene have led to the classification of PCV2 strains into eight genotypes, ranging from PCV2a to PCV2h. Among these, PCV2a, PCV2b, and PCV2d represent the primary phenotypes [13,14]. For example, a study by Yang et al. (2022) reported a PCV2 positivity rate of 26.46% among 257 samples from 23 pig farms in Southwest China, with the PCV2d genotype being the most prevalent [15]. Additionally, Jia et al. (2022) reported an even higher positive rate of 72.90% from 1760 clinical samples collected in Henan Province from 2018–2019 [16]. Similar patterns were noted in Northeast China, where Xia et al. (2019) reported a positivity rate of 50.0% among 472 domestic pig samples, with PCV2d accounting for 60.6% of the cases [17]. A recent nationwide epidemiological investigation in China (2024) further reported a high overall PCV2 positivity rate of 71%, with PCV2d remaining the dominant genotype across various regions, including those neighboring Hubei [18].

Commercial vaccines for PCV2 have been thoroughly validated and shown to control clinical disease effectively. This is reflected in a reduced incidence of PCV2 systemic disease (PCV2-SD), improved daily weight gain, decreased viremia, reduced viral shedding in feces, and diminished coinfections, all of which increase overall farm productivity [19,20,21]. The first PCV2 vaccines, including inactivated and subunit types, were introduced in Europe in 2004 and North America in 2006 [3,22]. The optimal window for vaccination is typically between 3 and 6 weeks of age; delaying immunization until 8 to 10 weeks may allow viremia to persist until 18 to 25 weeks of age [23]. Cross-protection among vaccines targeting different genotypes is generally strong [21,24], with bivalent vaccines (PCV2a-PCV2b) offering superior immunity compared with monovalent options [25].

Several commercial PCV2 vaccines are currently available on the international market, including Circovac^®^ (Merial, Lyon, France), Fostera™ PCV (Zoetis, Parsippany, NJ, USA), Ingelvac CircoFLEX^®^ (Boehringer Ingelheim, Ingelheim am Rhein, Germany), Circumvent (MSD Animal Health, Madison, NJ, USA), and Porcilis PCV^®^ (MSD Animal Health, Madison, NJ, USA). These vaccines have consistently demonstrated significant efficacy in mitigating the clinical manifestations of porcine circovirus-associated disease and substantially improving the growth performance of swine. Within the Chinese market, ten commercially available PCV2 vaccines were documented in a study by Guo et al. [3]. These vaccines exhibit considerable heterogeneity in their genotype composition, design strategies, and routes of administration. Ingelvac CircoFLEX^®^, a commercial PCV2 subunit vaccine, employs the PCV2 ORF2 protein as its immunogenic component, adjuvanted with a carbomer. A case study conducted on an organic pig farm in the Netherlands revealed that vaccination with Ingelvac CircoFLEX^®^ resulted in a significant increase in the average daily weight gain (ADG) of weaned and fattening pigs by 21 g, alongside a 3.6% reduction in mortality [26]. These findings underscore its substantial positive impact on production indices and the mitigation of economic losses. Notably, the CP08 strain vaccine, an emerging domestic PCV2 vaccine in China (Chopper Biology Co., Ltd., Wuhan, China), currently lacks published data regarding its clinical efficacy. Consequently, this study was specifically designed to evaluate both the Ingelvac CircoFLEX^®^ and the CP08 strain vaccines in animal experiments. This investigation aims not only to validate the practical effectiveness of PCV2 vaccines in large-scale field trials within pig farms but also, critically, to assess the clinical efficacy of this novel domestic vaccine and elucidate any differences in performance compared with established imported vaccine products.

Hubei Province is a prominent pig-producing region in China, and its swine industry plays a vital role in the local agricultural economy. The rapid expansion of large-scale pig farms has led to significant increases in herd size and pig production. However, the challenges posed by PCV2 and its genetic variations continue to impact swine health management in this area. To better understand the local prevalence of PCV2, this study collected blood samples from large-scale farms between 2022 and 2024 and conducted an epidemiological survey via fluorescent quantitative PCR and enzyme-linked immunosorbent assay (ELISA). Additionally, two widely used commercial PCV2 vaccines were evaluated in animal trials to assess their immunogenicity (serum antigen and antibody detection) and effects on farm productivity (body weight gain, mortality rate, and economic returns). A cost-benefit analysis was also performed, considering vaccine investment and economic returns, to identify optimal vaccination strategies under varying pricing scenarios. These findings offer scientific and economic insights to support vaccine selection and the development of PCV2 control strategies in Hubei and similar regions, ultimately promoting sustainable growth within the swine industry.

## 2. Materials and Methods

### 2.1. Sampling Strategy for Epidemiology Survey

**Figure 1:**
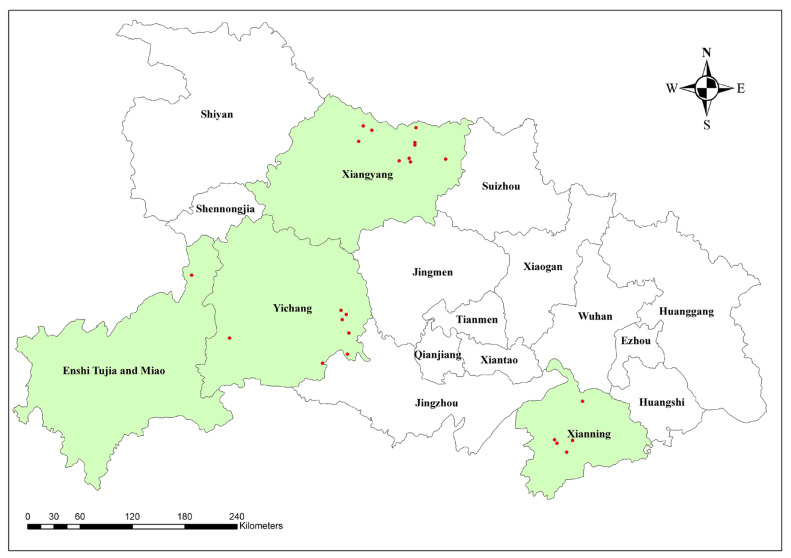
Sampling sites for PCV2 epidemiological survey in Hubei Province, 2022–2024. The green shaded areas represent the city regions where samples were collected, and the red dots indicate the specific locations of the pig farms.

From January 2022 to December 2024, samples were collected over three consecutive years from 24 “two-point” large-scale pig farms, encompassing breeding and associated rearing facilities, in Xiangyang, Yichang, Xianning, and Enshi in Hubei Province (Figure 1). These farms were selected based on their similar characteristics, including large-scale production (over 5000 pigs), standardized management protocols, and strict biosecurity measures. The primary pig breed on these farms was the Duroc × (Landrace × Yorkshire) crossbreed. Prior to this study, all farms implemented routine vaccination programs against PCV2 using various commercially available vaccines, but the effectiveness varied, prompting this investigation. In total, 6600 serum samples were obtained. Assuming an estimated prevalence (*p*) of 50%, a 95% confidence level (Z = 1.96), and a 10% margin of error (d), the minimum required sample size per farm was calculated to be 97 serum samples using the standard formula [27]. In practice, samples were stratified and randomly collected at a 6:1:1:1:1 ratio across various groups: sows, 4-week-old weaned piglets, 8-week-old nursery pigs, and 16-week-old and 20-week-old fattening pigs (Table 1). This approach resulted in 100 samples per farm, thus fulfilling the minimum sample size criteria [28]. To avoid the confounding effect of routine vaccination on serological results, the epidemiological survey exclusively utilized qPCR for PCV2 antigen detection to assess active viremia. The PCV2 ELISA antibody detection method was reserved for the subsequent vaccine efficacy trial to monitor the specific immune response post-vaccination. The collected samples were stored at 4 °C and promptly transported to the laboratory to preserve their integrity and ensure accurate PCV2 antibody and antigen testing.

$$n=\frac{z^{2}\times p\times (1-p)}{d^{2}}$$

### 2.2. PCV2 Quantitative Real-Time PCR Antigen Detection

PCV2 fluorescent PCR detection kits were obtained from Hunan Guanmu Biotechnology Co., Ltd. (Changsha, China). Viral DNA/RNA extraction was performed via magnetic bead kits from Hunan Shengce Biotechnology Co., Ltd. (Changsha, China), in conjunction with the KingFisher Flex Purification System (Thermo Fisher Scientific, Waltham, MA, USA) for automated nucleic acid extraction. The fluorescent PCR amplification was conducted on a CFX96 Touch real-time PCR system (Bio-Rad Laboratories, Hercules, CA, USA). Nucleic acids were extracted via magnetic beads, where cell lysis allowed the release of nucleic acids that specifically bind to the hydroxyl groups on the magnetic beads. Magnetic rods facilitate bead adsorption, transfer, and release, followed by the elution of nucleic acids in a buffer solution [29]. The PCR mixtures were prepared according to the manufacturer’s instructions and included reaction buffer, enzyme mixture, internal controls, and a DNA template. The thermal cycling conditions included initial preincubation at 50 °C for 2 min, Taq polymerase activation at 95 °C for 2 min, and denaturation at 95 °C for 15 s, followed by annealing and extension at 60 °C for 30 s. This cycle was repeated for a total of 40 cycles. Changes in the fluorescence signal during amplification were monitored in real time to determine the presence of PCV2 on the basis of cycle threshold (Ct) values. The validity criteria and sample classification, based on the manufacturer’s instructions for the commercial kit, were established as follows: no Ct for the negative control; a positive control for Ct ≤35; samples with Ct ≤ 40 were deemed positive; otherwise, they were classified as negative.

### 2.3. PCV2 ELISA Antibody Detection

Antibody levels were assessed via a commercial PCV2-dCap-ELISA kit (JNT Jinnuo Diagnostics, Beijing, China). The procedures closely followed the manufacturer’s instructions: (1) remove the PCV2 antigen-coated microplates; (2) dilute samples at a ratio of 1:100 in a dilution plate; (3) add 100 μL of the diluted sample along with 100 μL of both undiluted negative and positive controls to the wells; (4) incubate at 25 °C for 30 min and then wash the wells three times with 300 μL of wash buffer; (5) add 100 μL of TMB (3,3′,5,5′-Tetramethylbenzidine) substrate and incubate at 25 °C for an additional 30 min, followed by washing as previously described; (6) add 50 μL of stop solution and measure the optical density (OD) at 450 nm; (7) calculate the sample S/P ratio via the formula: S/P = (sample OD − mean negative control OD)/(mean positive control OD − mean negative control OD). Samples with an S/P ratio ≥ 0.4 were deemed antibody positive, whereas those with an S/P ratio less than 0.4 were considered negative.

### 2.4. Evaluation of Commercial PCV2 Vaccine Efficacy in a Fattening Farm

This study phase focused on fattening pigs for two primary reasons: first, our epidemiological data identified the nursery-to-fattening transition as a high-risk period for PCV2 infection; second, growth performance during the fattening stage is a critical determinant of economic returns in commercial pig production. This animal trial was conducted at a modern fattening farm located in Xiangyang city, which had previously been included in an epidemiological survey. The farm has standardized pig housing that ensures optimal temperature, humidity, and ventilation. It features automated systems for feeding, drinking, and waste disposal, facilitating a scientifically managed approach to husbandry. Additionally, the farm maintains a veterinary clinic and laboratory fully equipped for health monitoring and sample analysis.

On 27 November 2022, 1200 twenty-one-day-old weaned seronegative and antigen-negative individuals for PCV2 were selected. Owing to early clinical signs such as emaciation, lameness, depression, and anorexia, 71 piglets were culled, resulting in the enrollment of 1129 piglets for the trial. Two commercial PCV2 subunit vaccines were evaluated: the recombinant baculovirus CP08 vaccine (Chopper Biology Co., Ltd., Wuhan, China) and the Ingelvac CircoFLEX^®^ vaccine (Boehringer Ingelheim Co., Ltd., Ingelheim, Germany). In a double-blind randomized controlled design, piglets were assigned to two vaccinated groups or one control group with balanced initial body weights and sex ratios [30]. The initial weight distribution of the piglets was carefully managed to ensure comparability between groups. Piglets were weighed, and those falling outside the primary weight range (4.32–10.32 kg) were excluded. The remaining piglets were then allocated to three groups, with weight ranges established based on standard deviations from the mean to ensure a normal distribution within each group (Table 2). The experimental groups received 1 mL intramuscular injections of either the CP08 or Ingelvac CircoFLEX^®^ vaccine at 21 days of age; the controls received equivalent volumes of saline.

Before immunization, all piglets were ear-tagged and weighed, and baseline data were recorded. After vaccination, the piglets were cohoused to mimic realistic production environments and raised for 164 days until they reached market weight. Mortality was monitored, with records made of the ear tag number, date, and weight at the time of death. Before the market, the piglets were reweighed to assess their final body weight, and the average daily gain was calculated. As described in the sampling schedule, blood samples (*n* = 20 per group) were collected at weeks 4, 8, 12, 16, 20, and 24 to detect the PCV2 antigen and antibodies. All animal experiments in this study complied with the animal welfare regulations of the Chinese Experimental Animal Center and the ethical standards set by the Ethics Committee of Huazhong Agricultural University (Approval No. HZAUSW-2022-0007).

### 2.5. Statistical Analysis

Data analyses were performed with XLSTAT 2022 (Addinsoft, Paris, France). The normality of the weight data was assessed via the Shapiro-Wilk test [31]. The average weight gain per head was analyzed across various vaccine immunization groups via Kruskal-Wallis nonparametric tests [32], followed by Dunn’s multiple comparisons for instances of significant results [33]. Pearson’s chi-square test was used to examine group mortality rate differences [34]. A chi-square test was also used to compare PCV2 antigen positivity rates among the four geographical locations in the epidemiological survey. No significant differences were found (*p* > 0.05), justifying the pooling of data for overall analysis. A *p*-value < 0.05 was considered statistically significant. Graphical visualizations were created via ggplot2 v3.5.0 and ArcGIS 10.7 (ESRI, Redlands, CA, USA) [35]. Cost–benefit analysis was conducted to evaluate the economic benefits. The revenue per pig was calculated based on the average final body weight multiplied by the local market price for live hogs at the time of sale (assumed at ¥15 CNY/kg). The net benefit per pig for each vaccinated group was then calculated by subtracting the vaccine cost from the additional revenue generated compared to the control group.

## 3. Results

### 3.1. Epidemiological Survey of PCV2 Antigen

**Figure 2:**
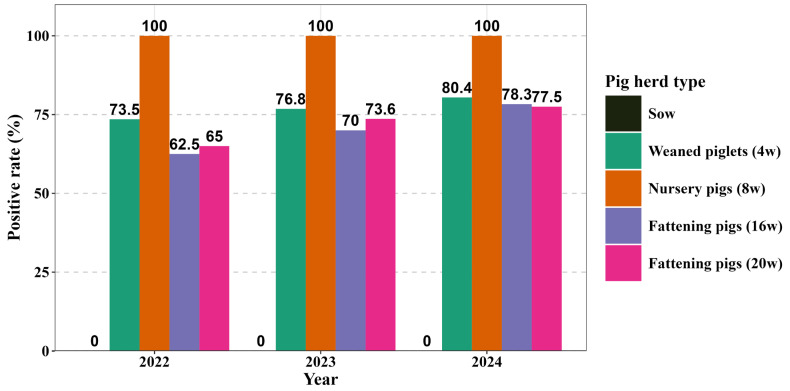
PCV2 antigen positivity rates by pig age group in Hubei Province, 2022–2024.

From 2022 to 2024, an epidemiological survey for PCV2 antigen was conducted across 24 large-scale pig farms in Hubei Province. The sample sizes included 2000 in 2022, 2200 in 2023, and 2400 in 2024, encompassing various age groups. Notably, no PCV2 antigen was detected in sows throughout the three-year study period, suggesting a low infection risk or adequate viral clearance at this developmental stage. In contrast, weaned piglets and fattening pigs presented high antigen positivity rates, with an observable increasing trend observed annually (see Figure 2). Remarkably, 8-week-old nursery pigs consistently demonstrated a 100% positivity rate, indicating that this age is the peak period for viral infection. The positivity rates for 4-week-old piglets increased from 73.50% in 2022 to 80.42% in 2024; for 16-week-old fattening pigs, the rates rose from 62.50% to 78.33%; and for 20-week-old fattening pigs, the rates increased from 65.00% to 77.50%. These results indicate that PCV2 transmission continues during the growing and finishing phases, suggesting the possibility of immunity gaps or vaccine failures outside the sow population.

### 3.2. Efficacy of Commercial Vaccines: Antigen and Antibody Dynamics

**Figure 3:**
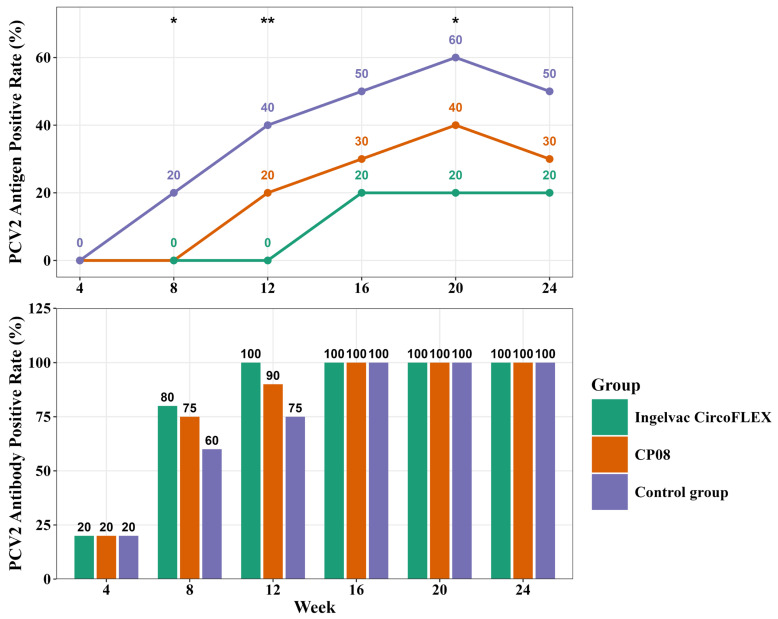
PCV2 antigen and antibody positive rates in vaccine trial groups over time. The figure is presented in two panels for clarity. (**Top** **panel**) PCV2 antigen positivity rates are shown as a line graph. (**Bottom** **panel**) PCV2 antibody positivity rates are shown as a bar chart. Statistical analysis was performed using Fisher’s exact test to compare positive rates among the three groups at each time point. Asterisks indicate significant differences: * *p* < 0.05, ** *p* < 0.01. Values above data points/bars represent the percentage of positive samples in each group.

At weeks 4, 8, 12, 16, 20, and 24, twenty blood samples were randomly collected from each group to evaluate the presence of PCV2 antigen and antibodies (refer to Figure 3). Across all groups, the antigen and antibody positivity rates increased with age. The Ingelvac CircoFLEX^®^-vaccinated group consistently exhibited significantly lower antigen positivity than the CP08- and control groups did, with the latter showing the highest rates. Notably, PCV2 antigen was first detected in the Ingelvac CircoFLEX^®^ group at 16 weeks (20%), whereas the CP08 and control groups presented earlier detection at 12 and 8 weeks, respectively. This finding indicates that vaccination effectively delays wild-type viral infection. By week 16, all groups reached 100% antibody positivity, with the Ingelvac CircoFLEX^®^ displaying slightly higher seropositivity than CP08 between weeks 4 and 12, while the control group recorded the lowest antibody rates.

### 3.3. Growth Performance and Mortality Analysis

**Figure 4:**
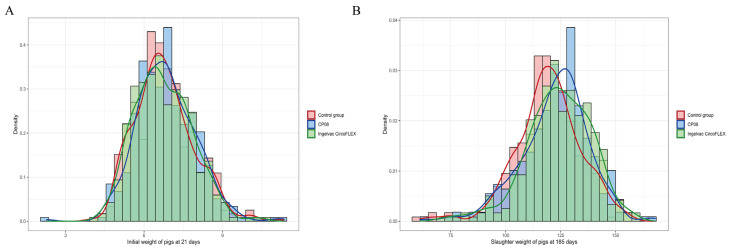
Body weight changes following PCV2 vaccination. The histograms display the frequency distribution of pig body weights for the Ingelvac CircoFLEX^®^, CP08, and Control groups. The different colored lines are the corresponding overlaid density curves, which illustrate the overall distribution trend for each respective group. The figure displays the distribution of pig body weights at the start and end of the trial: (**A**) Histogram and overlaid density curve showing the initial body weight distribution for 21-day-old weaned piglets. (**B**) Histogram and overlaid density curve showing the final market weight distribution after 164 days of rearing.

At the beginning of the trial, the piglets’ body weight distributions were assessed (Figure 4A). After 34 piglets that weighed less than 4.32 kg or greater than 10.32 kg during the normality screening, each group consisted of 365 piglets with average weights ranging from 6.76 to 6.78 kg, ensuring a comparable distribution across weight intervals (refer to Table 2). After 164 days of mixed-group rearing, the final weights and mortality rates were recorded (Table 3). Owing to mortality and ear tag loss, the final counts for weighing were 321 in the Ingelvac CircoFLEX^®^ group, 294 in the CP08 group, and 312 in the control group. A chi-square analysis revealed no significant differences in mortality rates among the groups (*p* = 0.732). Notably, the average daily weight gain was significantly greater in the Ingelvac CircoFLEX^®^ and CP08 groups than in the control group (*p* < 0.05), suggesting that PCV2 vaccination markedly enhances growth performance, regardless of the vaccine administered. Although the Ingelvac CircoFLEX^®^ group presented a slightly greater average weight gain (117.29 kg) than did the CP08 group (115.59 kg), this difference was not statistically significant (*p* > 0.05).

### 3.4. Economic Benefits of Commercial PCV2 Vaccines

Economic analyses based on average revenue per pig at market weight indicated returns of ¥2678.07, ¥2648.57, and ¥2590.95 for the Ingelvac CircoFLEX^®^, CP08, and control groups, respectively. Compared with the CP08 and control groups, Ingelvac CircoFLEX^®^ generated additional earnings of ¥29.50 and ¥87.12 per pig, respectively, whereas CP08 produced ¥57.62 more than the control group did (see Table 4). These results illustrate that commercial PCV2 vaccination enhances growth performance and offers significant economic advantages. The increased revenue in vaccinated groups compared to the control group was statistically significant (*p* < 0.05) based on the analysis of weight gain differences.

## 4. Discussion

This study presents a thorough epidemiological profile of PCV2 infection among various pig populations on large-scale farms in Hubei Province. The consistent absence of PCV2 antigen in sows indicates adequate viral clearance or a low risk of infection at this stage, which aligns with earlier studies that reported low viral loads and prolonged latency in mature sows [15,16]. Conversely, postweaning piglets and fattening pigs demonstrated an increasing trend in antigen positivity over time, with 100% positivity noted in 8-week-old nursery pigs. This finding indicates that this stage represents a peak in viral infection with a significant risk of transmission. These findings support the immune window hypothesis Oliver-Ferrando et al. (2016) proposed, underscoring the critical importance of early vaccination to prevent prolonged viremia and associated morbidity [23,36].

The two commercial PCV2 subunit vaccines demonstrated a significant reduction in antigen positivity, alongside increased daily weight gain and overall performance. These findings align with previous studies on vaccine efficacy by Chae (2012) and Segalés (2015) [19,20]. Notably, the Ingelvac CircoFLEX^®^ vaccine delayed antigen detection until 16 weeks and maintained a lower overall antigen positivity rate than the CP08 vaccine and control groups did, indicating superior viral control. This advantage may stem from broader antigenic coverage or an optimized adjuvant formulation, which supports Bandrick et al.’s (2022) assertion that bivalent vaccines are more effective than monovalent vaccines [25]. Recent field evaluations in 2024 further support this, demonstrating that a new PCV2d and Mycoplasma hyopneumoniae inactivated bivalent vaccine effectively reduces subclinical PCV2d infection and improves growth performance in pigs [37]. Furthermore, the observed cross-protection among various genotypes, as highlighted by Jeong et al. (2015) and Opriessnig et al. (2017), is consistent with our findings of improved growth performance across vaccinated groups, despite variations in magnitude [6,21].

Economically, despite higher costs, Ingelvac CircoFLEX^®^ demonstrated more substantial weight gain benefits and superior cost-effectiveness than did CP08. This highlights the importance of considering cost and performance gains when selecting vaccines for contemporary pig farming operations. This finding is particularly relevant as recent studies in 2024 have highlighted the frequency of PCV2 viremia in nursery piglets as a significant economic threat, even in integrated systems, due to subclinical infections leading to reduced productivity [38]. Additionally, mortality rates did not significantly differ among the groups, suggesting that vaccines primarily increase growth and reduce viral loads rather than directly influencing survival, which aligns with findings from previous studies [19,21].

In summary, this study clarifies the epidemiology of PCV2 on large-scale farms in Hubei and demonstrates the effectiveness of commercial vaccines. Future research should investigate the influence of PCV2 genetic variation on vaccine efficacy, the duration of vaccine-induced immunity, and the mechanisms of cross-genotype protection. Optimizing vaccination protocols, especially by implementing targeted immunization around weaning, could help close immunity gaps and reduce viral transmission, ultimately enhancing control strategies. This study is subject to several limitations. First, the absence of genetic sequencing on PCV2-positive samples precluded the identification of prevalent genotypes (e.g., PCV2d, PCV2b). This, in turn, limited our ability to assess the antigenic concordance between circulating field strains and the vaccine strains. Second, while the vaccine trial specifically used seronegative animals, the broader epidemiological survey did not quantify maternally derived antibody (MDA) levels, which could influence early infection dynamics and the determination of the optimal vaccination window. Third, our sampling was restricted to large-scale, modern pig farms; therefore, the findings may not be generalizable to smaller farms with different management and biosecurity practices. Finally, the evaluation of vaccine efficacy was confined to production parameters and viremia, and did not include an analysis of cellular immunity, which is a critical component for the clearance of PCV2 infection.

## 5. Conclusions

This study systematically examined the epidemiology of PCV2 in large-scale pig farms throughout Hubei Province from 2022–2024, revealing distinct infection patterns at various production stages. Notably, the absence of PCV2 antigen in sows contrasted with the high and increasing positivity rates observed in weaned and fattening pigs, particularly among 8-week-old nursery pigs. This highlights critical windows for virus transmission and potential gaps in immunity. The evaluation of two widely utilized commercial PCV2 subunit vaccines demonstrated their effectiveness in reducing viral antigen prevalence and enhancing growth under field conditions. Ingelvac CircoFLEX^®^ showed superior viral control and offered economic advantages. These findings offer valuable insights for optimizing PCV2 control strategies and vaccine selection in Hubei and similar regions. Ongoing monitoring of PCV2 epidemiology, coupled with strategically timed vaccinations and estimation of economic costs, is essential for maintaining vaccine efficacy and promoting the health and productivity of swine populations. This research provides a solid foundation for evidence-based decision-making toward the sustainable development of the swine industry.

## Figures and Tables

**Table 1:** Number of pig farms sampled and PCV2 antigen detection sample counts.

Year	Number of Pig Farms	Pig Herd Type					Total
		Sow	Weaned Piglets (4 Weeks)	Nursery Pigs (8 Weeks)	Fattening Pigs (16 Weeks)	Fattening Pigs (20 Weeks)	
2022	20	1200	200	200	200	200	2000
2023	22	1320	220	220	220	220	2200
2024	24	1440	240	240	240	240	2400
Total	66	3960	660	660	660	660	6600

**Table 2:** Distribution of pig numbers by weight range across groups before trial initiation.

Group	Weight (kg)						Total (Head)	Average Weight (kg)
	4.32–5.32	5.32–6.32	6.32–7.32	7.32–8.32	8.32–9.32	9.32–10.32		
Ingelvac CircoFLEX^®^	26	107	122	80	29	1	365	6.76
CP08	24	99	138	81	23	0	365	6.78
Control group	31	99	137	62	34	2	365	6.77
Total (head)	81	305	397	223	86	3	1095	6.77

**Table 3:** Mortality rates and final weight gain per group following 164 days of feeding.

Group	Number of Pig Missing Ear Tags	Number of Pig Deaths	Mortality Rate (%)	χ^2^ Values	*p* Value ^a^	Weighing Head Number	Average Weight Gain per Head (kg) (Mean ± Sd) ^b^
Ingelvac CircoFLEX^®^	23	21	6.14	5.991	0.732	321	117.29 ± 14.70 ^a^
CP08	45	26	8.12			294	115.59 ± 15.02 ^a^
Control group	28	25	7.42			312	112.65 ± 14.75 ^b^

Note: ^a^ Mortality rates were analyzed using Pearson’s chi-square test. ^b^ Average daily weight gain was assessed using the Kruskal–Wallis nonparametric test followed by Dunn’s multiple comparisons. Different superscript letters indicate statistically significant differences between groups.

**Table 4:** Comparative average revenue per pig at market weight across groups.

Group	Vaccine (¥/Head)	Price (¥/Head) ^a^	Average Revenue (¥/Head) ^b^	Difference Among Groups (¥/Head)		
				Ingelvac CircoFLEX^®^ vs. COP08	Ingelvac CircoFLEX^®^ vs. Control Group	CP08 vs. Control Group
Ingelvac CircoFLEX^®^	19.6	23	2678.07	29.5	87.12	57.62
CP08	10		2648.57			
Control group	0		2590.95			

Note: ¥: Chinese Yuan (CNY). ^a^ National average pork price in April 2025, sourced from the Ministry of Agriculture and Rural Affairs of China. ^b^ Calculation formula of average revenue: Average weight gain × Price per kilogram − Vaccine cost.

## Data Availability

The data presented in this study are available within the article. The raw data supporting the conclusions of this article are available from the corresponding author upon reasonable request.

## Abbreviations

The following abbreviations are used in this manuscript:

PCV2	Porcine circovirus type 2
Ct	Cycle threshold
OD	Optical density
PCV-AD	Porcine circovirus-associated disease
PCV2-SD	Porcine circovirus type 2 systemic disease
ORF	Open reading frames
ADG	Average daily weight gain
ELISA	Enzyme-linked immunosorbent assay
PMWS	Postweaning multisystemic wasting syndrome
TMB	3,3′,5,5′-Tetramethylbenzidine

## References

[1] Segalés J., Kekarainen T., Cortey M. (2013). The natural history of porcine circovirus type 2: From an inoffensive virus to a devastating swine disease?. Vet. Microbiol..

[2] Harding J.C.S. (2004). The clinical expression and emergence of porcine circovirus 2. Vet. Microbiol..

[3] Guo J., Hou L., Zhou J., Wang D., Cui Y., Feng X., Liu J. (2022). Porcine circovirus type 2 vaccines: Commercial application and research advances. Viruses.

[4] Yu C., Cao M., Wei Y., Liu J., Zhang H., Liu C., Feng L., Huang L. (2023). Evaluation of cross-immunity among major porcine circovirus type 2 genotypes by infection with PCV2b and PCV2d circulating strains. Vet. Microbiol..

[5] Segalés J., Domingo M. (2002). Postweaning multisystemic wasting syndrome (PMWS) in pigs. A review. Vet. Quart..

[6] Opriessnig T., Meng X.J., Halbur P.G. (2007). Porcine circovirus type 2 associated disease: Update on current terminology, clinical manifestations, pathogenesis, diagnosis, and intervention strategies. J. Vet. Diagn. Invest..

[7] Finsterbusch T., Mankertz A. (2009). Porcine circoviruses—Small but powerful. Virus Res..

[8] Tischer I., Gelderblom H., Vettermann W., Koch M.A. (1982). A very small porcine virus with circular single-stranded DNA. Nature.

[9] Nayar G.P., Hamel A., Lin L. (1997). Detection and characterization of porcine circovirus associated with postweaning multisystemic wasting syndrome in pigs. Can. Vet. J..

[10] Hamel A.L., Lin L.L., Nayar G.P. (1998). Nucleotide sequence of porcine circovirus associated with postweaning multisystemic wasting syndrome in pigs. J. Virol..

[11] Karuppannan A.K., Opriessnig T. (2017). Porcine Circovirus Type 2 (PCV2) Vaccines in the Context of Current Molecular Epidemiology. Viruses.

[12] Franzo G., Cortey M., Segales J., Hughes J., Drigo M. (2016). Phylodynamic analysis of porcine circovirus type 2 reveals global waves of emerging genotypes and the circulation of recombinant forms. Mol. Phylogenet. Evol..

[13] Firth C., Charleston M.A., Duffy S., Shapiro B., Holmes E.C. (2009). Insights into the evolutionary history of an emerging livestock pathogen: Porcine circovirus 2. J. Virol..

[14] Xiao C.T., Halbur P.G., Opriessnig T. (2015). Global molecular genetic analysis of porcine circovirus type 2 (PCV2) sequences confirms the presence of four main PCV2 genotypes and reveals a rapid increase of PCV2d. J. Gen. Virol..

[15] Yang Y., Xu T., Wen J., Yang L., Lai S., Sun X., Xu Z., Zhu L. (2022). Prevalence and phylogenetic analysis of porcine circovirus type 2 (PCV2) and type 3 (PCV3) in the Southwest of China during 2020–2022. Front. Vet. Sci..

[16] Jia Y., Zhu Q., Xu T., Chen X., Li H., Ma M., Zhang Y., He Z., Chen H. (2022). Detection and genetic characteristics of porcine circovirus type 2 and 3 in Henan province of China. Mol. Cell. Probes.

[17] Xia D., Huang L., Xie Y., Zhang X., Wei Y., Liu D., Zhu H., Bian H., Feng L., Liu C. (2019). The prevalence and genetic diversity of porcine circovirus types 2 and 3 in Northeast China from 2015 to 2018. Arch. Virol..

[18] Yang K., Wang Z., Wang X., Bi M., Hu S., Li K., Pan X., Wang Y., Ma D., Mo X.J.V.J. (2024). Epidemiological investigation and analysis of the infection of porcine circovirus in Xinjiang. Virol. J..

[19] Chae C. (2012). Commercial porcine circovirus type 2 vaccines: Efficacy and clinical application. Vet. J..

[20] Segalés J. (2015). Best practice and future challenges for vaccination against porcine circovirus type 2. Expert Rev. Vaccines.

[21] Jeong J., Park C., Choi K., Chae C. (2015). Comparison of three commercial one-dose porcine circovirus type 2 (PCV2) vaccines in a herd with concurrent circulation of PCV2b and mutant PCV2b. Vet. Microbiol..

[22] Ellis J. (2014). Porcine circovirus: A historical perspective. Vet. Pathol..

[23] Oliver-Ferrando S., Segalés J., López-Soria S., Callén A., Merdy O., Joisel F., Sibila M. (2016). Evaluation of natural porcine circovirus type 2 (PCV2) subclinical infection and seroconversion dynamics in piglets vaccinated at different ages. Vet. Res..

[24] Opriessnig T., Xiao C.T., Halbur P.G., Gerber P.F., Matzinger S.R., Meng X.J. (2017). A commercial porcine circovirus (PCV) type 2a-based vaccine reduces PCV2d viremia and shedding and prevents PCV2d transmission to naïve pigs under experimental conditions. Vaccine.

[25] Bandrick M., Balasch M., Heinz A., Taylor L., King V., Toepfer J., Foss D. (2022). A bivalent porcine circovirus type 2 (PCV2), PCV2a-PCV2b, vaccine offers biologically superior protection compared to monovalent PCV2 vaccines. Vet. Res..

[26] Schlepers M., Gelauf J., Wertenbroek N., Nielen M. (2013). Improvement of technical results following use of Ingelvac CircoFLEX in a Dutch organic breeding and fattening farm: A case report. Tijdschr. Voor Diergeneeskd..

[27] Thrusfield M. (2018). Veterinary Epidemiology.

[28] Fleiss J.L., Levin B., Paik M.C. (2013). Statistical Methods for Rates and Proportions.

[29] Haukanes B.-I., Kvam C. (1993). Application of magnetic beads in bioassays. Biotechnology.

[30] Festing M.F., Altman D.G. (2002). Guidelines for the design and statistical analysis of experiments using laboratory animals. ILAR J..

[31] Shapiro S.S., Wilk M.B. (1965). An analysis of variance test for normality (complete samples). Biometrika.

[32] McKight P.E., Najab J. (2010). Kruskal-Wallis Test.

[33] Dunn O.J., Clark V.A. (2009). Basic Statistics: A Primer for the Biomedical Sciences.

[34] Pearson K.X. (1900). On the criterion that a given system of deviations from the probable in the case of a correlated system of variables is such that it can be reasonably supposed to have arisen from random sampling. Lond. Edinb. Dubl. Phil. Mag..

[35] Villanueva R.A.M., Chen Z.J. (2019). ggplot2: Elegant Graphics for Data Analysis. Meas. Interdiscip. Res. Perspect..

[36] Fan M., Bian L., Tian X., Hu Z., Wu W., Sun L., Yuan G., Li S., Yue L., Wang Y. (2023). Infection characteristics of porcine circovirus type 2 in different herds from intensive farms in China, 2022. Front. Vet. Sci..

[37] Ham S., Suh J., Kim C., Seo B.J., Park G.S., Chae C.J.V.M. (2024). A field evaluation of a new porcine circovirus type 2d and Mycoplasma hyopneumoniae bivalent vaccine in herds suffering from subclinical PCV2d infection and enzootic pneumonia. Vet. Med. Sci..

[38] Sagrera M., Garza-Moreno L., Sibila M., Oliver-Ferrando S., Cárceles S., Casanovas C., Prieto P., García-Flores A., Espigares D., Segalés J.J.P.h.m. (2024). Frequency of PCV-2 viremia in nursery piglets from a Spanish swine integration system in 2020 and 2022 considering PRRSV infection status. Porc. Health Manag..

